# Effectiveness and Consequences of Direct Access in Physiotherapy: A Systematic Review

**DOI:** 10.3390/jcm12185832

**Published:** 2023-09-07

**Authors:** Marco Gallotti, Benedetta Campagnola, Antonello Cocchieri, Firas Mourad, John D. Heick, Filippo Maselli

**Affiliations:** 1 Catholic University of the Sacred Heart, Rome Campus, 00168 Rome, Italy; 2 University Hospital Foundation Campus Bio-Medico, Rome University, 00128 Rome, Italy; 3 Department of Physiotherapy, LUNEX International University of Health, Exercise and Sports, 4671 Luxembourg, Luxembourg; 4 Luxembourg Health & Sport Sciences Research Institute A.s.b.l., 50, Avenue du Parc des Sports, 4671 Luxembourg, Luxembourg; 5 Department of Physical Therapy, Northern Arizona University, P.O. Box 15105, Flagstaff, AZ 86011, USA; 6 Department of Human Neurosciences, Sapienza University of Rome, 00185 Rome, Italy

**Keywords:** direct access, physical therapy, self-referral, primary care, cost-effectiveness, physiotherapy

## Abstract

**Background.** Direct access in physiotherapy (DAPT) occurs when a patient has the ability to self-refer to physical therapy without physician referral. This model of care in musculoskeletal diseases (MSDs) has shown better outcomes than the traditional-based medical model of care that requires physician referral to access physiotherapist services. This traditional physician referral often results in a delay in care. Unfortunately, DAPT is still not permitted in many countries. **Objectives.** The primary objective of this systematic review was to compare the effectiveness, safety, and the accuracy of DAPT compared to the physician-led model of care for the management of patients with musculoskeletal disorders. The secondary objective of the present study is to define the physiotherapists’ characteristics or qualifications involved in DAPT. **Materials and methods.** Databases searched included: Medline, Scopus and Web of Science. Databases were searched from their inception to July 2022. Research strings were developed according to the PICO model of clinical questions (patient, intervention, comparison, and outcome). Free terms or synonyms (e.g., physical therapy; primary health care; direct access; musculoskeletal disease; cost-effectiveness) and when possible MeSH (Medical Subject Headings) terms were used and combined with Boolean operators (AND, OR, NOT). Risk of bias assessment was carried out through Version 2 of the Cochrane risk-of-bias tool (ROB-2) for randomized controlled trials (RCTs) and the Newcastle Ottawa Scale (NOS) for observational studies. Authors conducted a qualitative analysis of the results through narrative analysis and narrative synthesis. The narrative analysis was provided for an extraction of the key concepts and common meanings of the different studies, while the summary narrative provided a textual combination of data. In addition, a quantitative analysis was conducted comparing the analysis of the mean and differences between the means. **Results.** Twenty-eight articles met the inclusion criteria and were analyzed. Results show that DAPT had a high referral accuracy and a reduction in the rate of return visits. The medical model had a higher use of imaging, drugs, and referral to another specialist. DAPT was found to be more cost-effective than the medical model. DAPT resulted in better work-related outcomes and was superior when considering patient satisfaction. There were no adverse events noted in any of the studies. In regard to health outcomes, there was no difference between models. ROB-2 shows an intermediate risk of bias risk for the RCTs with an average of 6/9 points for the NOS scale for observational studies. **Conclusion.** DAPT is a safe, less expensive, reliable triage and management model of care that results in higher levels of satisfaction for patients compared to the traditional medical model. Prospero Registration Number: CRD42022349261.

## 1. Introduction

Musculoskeletal disorders (MSDs) are injuries or disorders affecting the body movement or the musculoskeletal system [1]. MSDs are the second most common cause of disability worldwide [2], with a prevalence comparable to the sum of all cardiovascular and chronic respiratory diseases. MSDs resulted in an economic burden of more than USD 800 billion (US) in 2015 [3]. Due to the significant impact of MSDs, it is essential to consider value-based care and work towards examining new approaches to manage MSDs more efficiently. Direct access to physiotherapy (DAPT) occurs when a patient can self-refer to a physiotherapist without having to see another health professional for a medical prescription [4]. Preliminary evidence suggests that DAPT could offer a promising option compared to other traditional models of care, particularly the physician-led model of care [5,6,7,8,9]. The effectiveness of DAPT has been reported by authors in several areas: reduction in direct and indirect costs for the patient and the national health system [6], reduction in work overload for general practitioners (GPs) [7], and improvement in health indicators for patients (e.g., health-related quality of life, quality-adjusted life years) [9]. World Physiotherapy itself, a global organization that represents the profession of physiotherapists at an international level, advocates for the growing responsibilities of the profession. This phase shift for the profession of physiotherapy is why clarifying the efficacy and safety of DAPT for patients with musculoskeletal disorders is needed [4,10,11,12,13,14]. Therefore, the clinical question explored in this manuscript is whether DAPT is safe, accurate, cost-effective, and does it socially impact the episode of care for the patient compared to the physician-led model of care for patients suffering from MSDs. The secondary objective of the present study is to define the physiotherapists’ characteristics or qualifications involved in DAPT. The study was conducted following the criteria of the 2020 PRISMA model.

## 2. Material and Methods

This SR was conducted following the updated Preferred Reporting Items for Systematic reviews and Meta-analyses (PRISMA) 2020 Statement [15].

**Protocol and Registration**

The SR protocol was prospectively registered on the International Prospective Register of Systematic Reviews (PROSPERO) on 6 August 2022 (registration number CRD4202234926).

**Eligibility criteria**

Primary studies (i.e., observational studies and randomized controlled trials) that dealt with the evaluation of DAPT management accuracy, cost-effectiveness, work-related outcomes, safety, patient satisfaction, and health outcomes were selected and compared with the physician-led model of care. Management accuracy was defined as the ability of physiotherapists to independently assess a patient without additional medical consultation. Cost-effectiveness was defined as the degree of efficacy and productivity in relation to its costs in the management of patients affected by MSDs. Work-related outcomes were intended as the comparison of the impact of the MSD on the patient’s occupational ability such as sick days or medical leave. Patient satisfaction was assessed as the quality of care perceived by the patients while safety was measured as the number and severity of adverse events. Health outcomes were the results of both physician-led and physiotherapist-led interventions measured through quantitative scales such as self-reported outcomes. Waiting times to receive the intervention were considered as a health outcome as the precocity of the intervention, both in evaluation and management, has a direct correlation with important patient health outcomes [16,17,18,19]. Inclusion and exclusion criteria are reported in Table 1.

### 2.1. Literature Search

The authors searched Medline, Scopus, and Web of Science databases from July 1996 to July 2022. According to the Peer Review of Electronic Search Strategies (PRESS) guideline statement [20], the authors generated a search string to assess the quality and comprehensibility of the literature search that was reviewed by two reviewers (MG and FM). Any disagreements that arose between the primary two reviewers was resolved through discussion or with a third reviewer (AC). Search terms describing DAPT, MSDs, and outcome measures of interest were combined to create a search string of appropriate words. The MEDLINE search strategy was developed using the PICO strategy for the development of the clinical question with the addition of medical subject headings (MeSH). MeSH and all relevant free-text words were combined using the Boolean operators (e.g., AND, OR). Table 2 summarizes the search terms and strategy for Medline.

**Study selection and data collection process**

Two reviewers (MG and FM) independently screened titles and abstracts. Discrepancies were discussed and considered by both reviewers and another independent reviewer (AC) was consulted if the disagreement was not solved. Subsequently, full text articles were assessed for eligibility and the reasons were recorded in the event of exclusions. The authors used the Rayyan software platform to organize and manage the selection process. 

All reviewers (MG, FM, AC, BC, FM, JH) independently extracted and included data for DAPT and for the physician-led model of care in a standardized extraction sheet that included: the first author’s full-name, year of publication, type of study, patient characteristics (age, gender, and type of MSDs), the management accuracy (i.e., assessed as the percentage of patients independently screened by the physiotherapist without additional medical consultation), cost-effectiveness (number of visits, imaging, and lost working days), safety (assessment of the number of adverse events), patient satisfaction (satisfaction questionnaires, Likert scale of satisfaction, interviews and questionnaires about quality perceived), and health outcomes (condition specific questionnaires, quality of life, perception of disability, psychological health status, patient’s coping, pain-related catastrophization, pain using the Visual Analogical Scale (VAS) and Numeric Pain Rating Scale (NPRS)). 

Data were extracted in parallel by two authors (MG and AC) to reduce the risk of bias [21].

**Data synthesis**

Outcomes analyzed by this review were qualitative as well as quantitative. After the data extraction was completed, a single reviewer (MG) grouped all studies that focused on a specific outcome of interest in the present review and then summarized the results by presenting the key concepts and elements most shared among the studies through a textual description. This process was considered due to the heterogeneity of the included studies [21,22]. In addition, whenever possible, a quantitative analysis was conducted. In regard to conducting a quantitative analysis for this systematic review, different approaches depending on the outcome of interest were conducted. For example, to describe the most common type of setting analyzed, authors described a numeric count. When the outcome of interest involved the comparison of the effects of DAPT in comparison with the medical model, the ranges, mean, and differences between the mean were used [21]. Tables and graphics were used to summarize data for visual explanation.

### 2.2. Risk of Bias Assessment

Risk of Bias (RoB) was assessed independently by two authors (MG and BC). The RoB 2 tool [23] was used for randomized controlled trials (RCTs) to consider all potential areas of bias to include: the randomization process, deviations from the intended interventions, missing outcome data, measurement of the outcome, and selection of the reported result. Furthermore, an overall risk-of-bias judgement was determined from using the RoB2 tool. Two authors reviewed every domain of the RCTs and judged the overall risk-of-bias or if the risk of bias was “low”, “some concerns”, or “high”. 

The NOS scale [24] for non-randomized studies was used for: retrospective case-control, observational studies, and prospective observational cohort studies. The scale comprises nine items investigating three domains: (i) sample selection (four points), (ii) comparability (two points), and (iii) outcome (three points) for case-control and cohort studies, respectively. A cut-off for methodological quality has not yet been validated for observational studies. However, although NOS does not allow for a quantitative score, each star attributable to a single item of the NOS could be considered as a point, with scores ranging between zero and nine for the NOS [25].

## 3. Results

**Figure 1:**
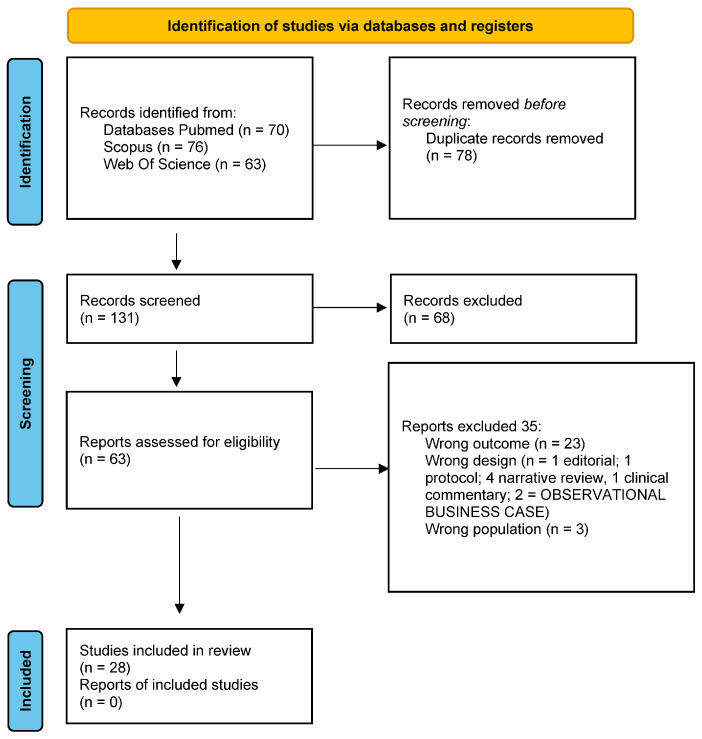
PRISMA flow diagram.

The study selection process and the included trials are reported in Figure 1. The authors initially identified a total of 209 articles. Seventy-eight articles were deleted as duplicates. A total of 103 articles were screened for title and abstract resulting in 103 articles being deleted as unsuitable due to not meeting the inclusion criteria. Of the 63 articles screened for full text, a total of 28 articles were included in the review. The 28 included studies were published from 1999 to 2022 [9,16,17,18,19,26,27,28,29,30,31,32,33,34,35,36,37,38,39,40,41,42,43,44,45,46,47,48]. The following study designs were identified: 10 RCTs, 9 prospective longitudinal cohort studies, 4 case control studies, 3 retrospective observational studies, and 2 prospective observational studies [9,16,17,18,19,26,27,28,29,30,31,32,33,34,35,36,37,38,39,40,41,42,43,44,45,46,47,48]. 

### 3.1. Population

A total of 32,742 patients underwent assessment and management through the DAPT care model, while 9900 patients underwent assessment and management through the physician-led care model, with a total number of 42,642 patients. Characteristics of the population are summarized in Table 3. Age, sex, and gender were not available for four studies [19,27,31,48]; seven studies did not provide additional details regarding the type and location of musculoskeletal disorder [19,27,28,31,32,36,43], while seventeen studies did not specify pathology onset [9,18,19,26,27,28,29,31,32,36,37,38,39,40,41,45,48]. 

All experimental interventions provided traditional DAPT, but two studies evaluated patients remotely by phone [34,36]. Characteristics and qualifications of the involved physiotherapists in DAPT are summarized in Table 4.

Seven studies [16,19,26,29,39,40,41] did not include a control. Interventions for the control group were delivered by a physician in eight studies [27,28,31,34,43,45,46,48]; by a general practitioner in eight studies [9,32,35,36,37,38,44,47], and by an orthopedic physician in five studies [17,18,30,33,42].

**Figure 2:**
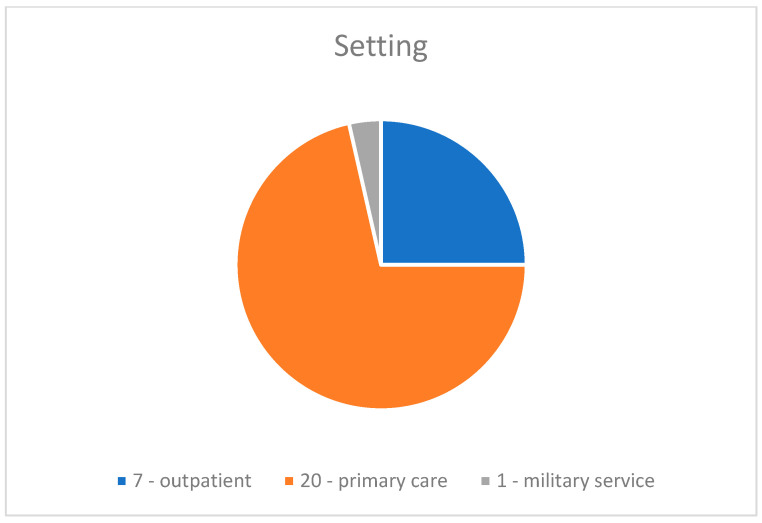
Setting of care.

The different setting of care is reported in Figure 2: eight studies were performed in an outpatient setting [16,19,26,34,41,42,43,46], one study in a military service setting [48], and twenty in a primary care hospital setting [8,9,17,18,27,28,29,30,31,32,33,35,36,37,38,39,40,44,45,47].

### 3.2. DAPT Management Accuracy

Fourteen studies evaluated the triage proficiency of physiotherapists [16,18,19,26,29,31,33,36,40,41,42,46,47,49]. Most commonly, physiotherapists triage patients without additional medical consultation [16,18,19,26,31,36,40,41,42,46,47]. Three studies compared the surgical conversion rate expressed as a percentage (i.e., surgical conversion rate is considered a useful measure of appropriateness of referrals, as it is a measure of the percentage of patients that were referred to a physician and underwent surgery) [19,40,41]. Referral selection accuracy was used as a measure of management accuracy only in one study [33]. The rate of return to visit or rate of re-referral was evaluated in four studies [29,31,33,47]. The agreement between physiotherapy and medical evaluation was evaluated in three studies [18,31,49]. Management accuracy is summarized in Table 5, resulting in secondary care referral accuracy (15% average difference) in favor of DAPT.

The return rate for further medical examination following physiotherapy discharge ranged between 0.9% and 9% (average 5.87%) from four studies without a control group [29,31,33,47]. Two studies estimated the agreement rate between physiotherapist and medical evaluation ranging between 74% and 87% (average 80.5%) [18,31]. Only one study evaluated the agreement of a chief radiologist toward physiotherapist prescription of radiographs for patients directly managed by physiotherapists [27]. In this study, the authors noted an agreement rate of 100% for validity, clarity, and appropriateness of physiotherapist prescription [27].

### 3.3. Cost-Effectiveness

Sixteen studies evaluated cost-effectiveness between models using different outcomes [7,9,27,28,31,33,34,35,36,37,42,43,44,45,47,48]:

- Use of health system resources, calculating the intervention costs: medication use and number of imaging referrals [9,27,31,33,34,35,37,42,45,46,47,48];
- Cost sustained by the patient [27,34,46];
- Patient savings [36,48];
- Incremental Cost Effectiveness Ratio (ICER) [9,43,45];
- Benefits estimated by the Quality-Adjusted Life Years (QALYs) [9,34,43,45];
- Time needed to deliver the triage process [28];
- Waiting time rate per visit [32,34];
- Timeframe before discharge [28];
- Number of visits needed for discharge [44].

Health system resources use was found to be the most widely used method among the included studies to estimate cost-effectiveness [9,27,31,33,34,35,37,42,45,46,47,48]. 

Intervention types commonly prescribed in the included studies are summarized in Table 6.

DAPT resulted in EUR 39.370 and EUR 62.867 of savings [9,27,35] in a timeframe ranging from 6 months [27] to 1 year [9,35]. A summary of compared wage costs and treatment options [42,45] are reported in Table 7. 

One study found no significant differences between the total savings for the healthcare system between models [34], while another one found no statistically significant difference across the average costs of professionals [46].

ICER, or the incremental cost-effectiveness ratio, is a synthetic measure that represents the economic value of an intervention, compared with an alternative intervention. Three studies have shown the ICER to be smaller for DAPT, meaning that physiotherapist-led management is less expensive and more effective than a physician-led model of care [9,43,45].

Two studies did not find statistically significant differences for direct costs sustained by the patient between care models [34,46], while another study estimated a favorable cost for the patient who carries out DAPT equal to EUR 29.5 per visit compared to the medical model which had an expense of EUR 63.8, for a total cost saving to the patient equal to EUR 34.3 in favor of DAPT [27]. Two studies estimated between USD 36.42 and USD 129 saved per patient per episode of care in favor of DAPT [36,48], with an average of USD 82.71 saved favoring DAPT. Mallett et al. also calculated an amount of £84,387.80 and £124,472.06 as the projection of the total savings over a year for a physiotherapist-led service, towards a general practitioner (GP)-led pathway, initiated by telephone contact from the patient, followed by a subsequent telephone triage appointment with a physiotherapist [36]. Notably, the DAPT pathway ensures an increase of 0.07 and 0.047 (average 0.05) of QALYs [9,34,43,45]. Although not statistically significant, Bornhöft et al. agree that DAPT has the potential to be a better cost-effective option (9). 

Regarding the rate of presence to visit (i.e., the effective presence to visit of a patient after a phone scheduled appointment) results [34,36] are summarized in Table 8. 

Interestingly, the lack of missed appointments due to patient no-show would allow the health system to save between £84,387.80 and £124,472.06 in one year [36]. As for the outcome of total time needed to triage and to discharge the patient, results are summarized in Table 9. 

### 3.4. Work Related Outcomes

The impact of DAPT on patient’s ability to work was evaluated in 10 studies [9,17,32,34,37,39,43,45,46,48] which focused on:

- Self-administered questionnaire on patients’ work productivity [46] or questionnaire intended to measure self-efficacy in the workplace [43];
- Percentage of patients prescribed sick days [37,45];
- Number of sick days [9,17,45,48];
- Labor participation measured on a 3-point scale (1 = did not return to work; 2 = returned to work with adaptations; or 3 = returned to work without adaptations) [39];
- Time off work directly reported by the patients [32,34] or the amount of sick hour leaves [9].

No major difference was found for work productivity between the care models [46]. However, one study (without control group) mentioned better work performance favoring DAPT [43]. Samsson et al. found no significant differences in days lost from work due to MSDs between the DAPT group and the physician-referred medical model [17]. One longitudinal observational study without a control group and with a 10-year follow-up, evaluated the ability of the DAPT (without control group) on the restriction to work participation, showing that out of 423 patients visited through DAPT, 168 patients (39.7%) did not return to work, 123 patients (29.1%) returned to work with adjustments, and 132 (31.2%) returned to work without adjustments [39]. Finally, both models showed similar results regarding lost working days (21.27) and lost working hours [9,32,34]. The comparison of sick leave and number of days lost from work is summarized in Table 10 [9,37,45]. 

### 3.5. Patient Satisfaction

Ten studies evaluated the patient, using:

- A 10- and 7-point Likert scale [7,42];
- Satisfaction questionnaires [16,18,27,31,33,36,47];
- Qualitative surveys [34].

Different satisfaction questionnaires were used:

- A questionnaire related to the satisfaction in care received [47];
- A modified and adapted questionnaire for assessing the quality of direct remote-access care (telephone) [36];
- The Quality from the Patient’s Perspective Questionnaire (QPP) [33];
- A patient satisfaction questionnaire and a physician satisfaction questionnaire related to how the physiotherapist performed the triage [18];
- The Perceived Improvement Evaluation (PIVAS) questionnaire [50] and the Deyo and Diehl (DD) [51] questionnaire [16];
- A questionnaire on patients’ experience of care [31];
- A questionnaire on patient satisfaction/dissatisfaction with being referred to another professional or additional diagnostic investigations (e.g., X-ray) [27].

Comparison of the results was not suitable because of the heterogeneity of the outcome measures. Of the ten studies reporting patient satisfaction, only one study [46] did not report significant differences between DAPT and the medical model of care. Another study found that DAPT resulted in higher quality of the perceived treatment for the following [42]:

- patient dissatisfaction with staff communication;
- patient dissatisfaction with the quality of treatment received;
- patient dissatisfaction with the facilities.

In addition, patients evaluated by a physiotherapist were more satisfied with the care received than those assessed or managed within the physician-led pathway [16,19,31,33,36,47]. Another study also evaluated the physician’s satisfaction regarding a physiotherapist-led service: reporting high level of satisfaction, with an average score of 1.9 on a scale ranging from 1 to 3 [18]. One study investigated patient satisfaction/dissatisfaction for additional imaging referral [27]. A total of 91% of patients reported being very satisfied with the referral and 84% reported being very happy with the feedback received from the physiotherapists about the need for further diagnostic investigation.

### 3.6. DAPT Safety

Five studies [27,32,34,38,46] investigated DAPT safety by the number of adverse events (i.e., unexpected events that occur following an intervention without evidence of causality). As an example, an increase in pain after a physiotherapist intervention occurred. Only one study reported the occurred severity of the adverse events that occurred by categorizing the event as none, mild, moderate, or severe [46]. DAPT safety is reported in Table 11.

### 3.7. Health Outcomes

Health outcomes were investigated in 15 studies [16,17,28,32,34,35,36,38,39,42,43,45,46,47,48].

- Six studies [28,32,33,34,36,48] estimated waiting time for assessment/management of DAPT toward a physician-led model of care, of which five were estimated in days [32,33,34,36,48] and one was estimated in minutes [28]. Results of DAPT waiting times are summarized in Table 12;

- Three studies evaluated health outcomes using district-specific questionnaires [39,42,43], but there was a large heterogeneity of questionnaires used between studies;
- Quality of life was assessed in 13 studies. The most-often used tool was the EuroQoL (EQ5D) [16,17,34,36,38,42,43,45,47], followed by the Short Form Health Survey 36 (SF-36) [16,42], SF-12 [43], and SF-10 [46];
- Perception of disability was evaluated in four studies through the Pain Disability Index (PDI) [17], the Disease Repercussions Profile [42], or the Measure Yourself Medical Outcomes Profile and global improvement [34]. Koojiman et al. [35] analyzed and compared the percentage of patients who achieved the expected outcomes between patients who underwent DAPT and those who went to the physiotherapist following medical referral;
- One study [38] assessed functional disability in ADLs with the Disability Rating Index (DRI) and the patient’s attitudes towards their musculoskeletal disorder through the Attitude Responsibility for Musculoskeletal disorders scale (ARM);
- Oostendorp et al. [39] evaluated the patient’s coping through the Pain Coping Inventory (PCI) and general health with the Global Perceived Effect (GPE);
- Two studies [38,42] assessed psychological health (35,39) through the Hospital Anxiety and Depression Scale;
- Five studies measured pain, three using the VAS (Visual Analogic Scale) [16,39,42], one the Numeric Pain Rating Scale (NPRS) [38], and another the 10-point Likert scale [46];
- Risk of chronicity of musculoskeletal pathology was carried out by two studies [38,43] through the Örebro Musculoskeletal Pain Screening Questionnaire (ÖMPSQ);
- Pain-related catastrophization of the patient was assessed by two studies [43,46] using the Pain Catastrophizing Scale (PCS);
- Two studies [39,43] evaluated avoidance behavior using the Fear Avoidance Belief Questionnaire (FABQ);
- Two studies [42,46] used the Pain Self Efficacy Questionnaire (PSEQ) to assess patient self-efficacy;
- One study [46] used the Patient-Specific Functional-Scale (PSFS) for physical function and the Patient Acceptability Symptom State (PASS) to measure acceptability of symptoms.

No significant differences between DAPT and the medical group were seen for all health outcomes except for quality-of-life assessment that showed contradictory evidence. Concerning quality of life, three studies reported the superiority of DAPT [33,34,38] and three studies noted no significant statistical difference [36,42,45].

### 3.8. Risk of Bias Assessment

**Figure 3:**
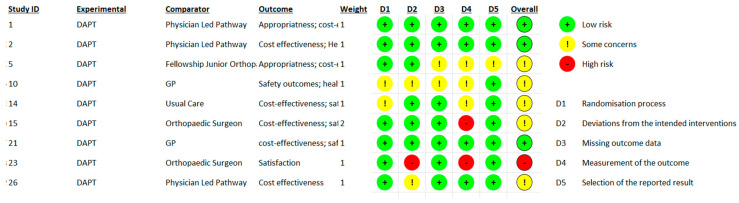
Rob-2 Score evaluation.

Overall, three studies were judged as demonstrating “low risk of bias” [32,45,46], six studies were rated as “some concern” [17,33,34,38,42], and one as “high risk” [30]. The Quality of RCT methodological evidence is summarized in Figure 3.

Even if the NOS scale does not allow for the estimation of a final score, each star was considered as a point to generate a score. Table 13 and Table 14 summarizes the NOS evaluation. For observational studies with a control group [18,28,35,36,37,43,44,47,48], the mean score was 6/9 points, with a minimum score of 4 and a maximum score of 9 points, while for observational studies without a control group [19,26,27,29,31,40,41,48] a mean score of 4.75/9 was reported.

## 4. Discussion

As an increasing number of countries have physiotherapists acting as entry level providers or assuming first contact roles, there is an increasing need for triage and differential diagnosis skills [52,53,54]. According to a previous review of the literature [55], the management accuracy of the physiotherapist working in a direct access setting was confirmed by the percentage of patients evaluated independently [16,18,19,26,31,36,40,41,42,46,47]. A high surgical conversion rate is considered a useful measure of appropriateness of DAPT referrals because it demonstrates the percentage of patients that were referred to a medical specialist who performs surgery [19,40,41]. Another indirect measurement of successful screening in DAPT is the rate of return for an additional visit after being discharged from therapy. In the current systematic review, a small percentage of patients needed additional consultations [29,31,33,47]. In addition, the appropriateness of evaluation was also observed to be high as measured by the agreement between physiotherapist and physician for both the following health action [56] and a referral for additional imaging [57]. All the above must be considered within the context of safe practice. The current study results found that DAPT is a safer model of care than the physician-led model, that was found to result in twice as many adverse events (3% versus 6% for the DAPT and physician-led model, respectively) [47,54,56,58,59]. DAPT safety has been found to be safer than the physician model of care in both longitudinal and retrospective studies [7,59]. Safety has been mentioned in a previous review [25], and the current study seems to confirm that the DAPT is a safe approach.

Cost-effectiveness is an important pillar for value-based care and provides strategies to implement models of care [60]. The results of the current study favor DAPT when compared to the physician-led model. One of the reasons could be explained by the different interventions offered between the two models. In DAPT, the most frequently used interventions are education, therapeutic exercise, manual therapy, and lifestyle management that requires the direct involvement of the patient. On the other hand, physicians’ interventions are dependent on physician actions and often require the use of technology, medication, or referral to a specialist physician [31]. Notably, a patient’s compliance in the health system must be considered as well. One example is the patient’s attendance rate to visits. Non-attendance (i.e., patients failing to appear for scheduled appointments) has consequences for clinic revenue because it may interrupt the flow of patients resulting in reduced productivity and lengthened waiting lists before receiving care [56]. The economic cost of a missed appointment results in a loss in supplier revenue due to a lower return than that achievable with the patient’s attendance at the visit [56]. According to the literature, the authors of the current study found that the economic burden related to a patient’s absenteeism for scheduled visits is less frequent in DAPT compared to physician-led pathways [56,61,62,63].

The cost-savings results of this review are in line with previous studies. A recent review demonstrated savings in favor of DAPT compared to the medical model [6] and this was confirmed by two specific economic reports [64,65]. These two studies confirmed the ability of DAPT to ensure similar health outcomes but through less costly, shorter, and fewer numbers of services compared to the medical model.

DAPT compared to the medical model has been reported to speed up the evaluation and ultimately led to a shorter episode of care for patients with MSDs than the medical model [28]. Other studies have demonstrated a better ability of other healthcare professionals in the management of primary care settings compared to the medical model, and this could probably also be extended to the DAPT [66,67]. The better work-related outcomes found in this review in favor of DAPT in terms of days lost for illness or injury (i.e., both requests or prescribed) [9,37,45] could be directly related to physiotherapists following clinical practice guidelines that recommend an active approach to MSDs [68], including interventions based on the early recovery of functional and job-related outcomes [38,42]. Education, reassurance, and a patient-centered approach are first-line interventions and are significant aspects of a holistic approach to the care of a person that may increase the perception of caretaking and result in the increased satisfaction reported by patients receiving DAPT [69,70,71]. Patients managed in DAPT have a shorter average waiting time [25,37,56,58,72] that positively impacts health outcomes and satisfaction [58,72,73]. No differences were found between health outcomes and quality of life between groups. Perhaps the absence of differences lies in the wide variety of quality of life and health outcomes and confounding variables such as psychosocial factors and duration of pain compared to what intervention is provided by the healthcare provider. These results seem to be in line with a recent review showing the absence of statistically significant differences between the two approaches regarding pain [6]. This review, in fact, showed similar improvement in the DAPT and medical model of care relating to pain suggesting no added benefit of direct access in reducing pain [6]. The results of the present review would also agree with a recent review which demonstrated the absence of significant differences from the point of view of PROMs related to pain and disability [55,68]. Both studies, in fact, underlined how the difference between direct access to physiotherapy and the medical model does not rely on health outcomes (PROMs or disability) but moreover on healthcare utilization costs.

Specific characteristics, training, or qualifications required by physiotherapists to perform DAPT do not exist. Studies included in the present review reported that experience in the musculoskeletal field and an “extended scope professional” (ESP) were important attributes for physical therapists performing DAPT (41). ESP is a physiotherapist qualification recognized as a professional working in an “extended” role after gaining specific training in the field of musculoskeletal physiotherapy and possessing clinical experience [59]. In any case, it is difficult to outline guidelines as to what training pathway or years of clinical experience a physical therapist should have to work in direct access. The heterogeneity of training pathways and the variability in their duration around the world (i.e., 3 years in Italy versus 5 years in England) makes it difficult to determine what needs to occur for a physical therapist to be able to work in a direct access environment. It would be useful to compare academic curriculum across the profession of physiotherapy at an international level to consider the appropriate training pathway and duration to answer this in a future study.

## 5. Limitations

According to a recent review [25], the overall quality of the evidence included in this review has an intermediate risk for bias for RCTs and an average score of 6 out of 9 points on the NOS scale [25]. Another limitation of this review is the heterogeneity of study design throughout the studies reviewed. The authors reviewed 10 RCTs, 9 prospective longitudinal cohort studies, 4 case control studies, 3 retrospective observational studies, and 2 prospective observational studies. Performance of a meta-analysis was not possible on these collected studies due to the heterogeneity of all included studies. Moreover, heterogeneity can also be seen in the outcome measures used to assess primary outcomes of this review (especially for patients’ satisfaction and health outcomes) that could not be merged through parametric statistical tools. Another aspect to consider is the length of time, July 1996 to July 2022, the databases were searched in the current study. During this period of 26 years, physiotherapy education has had many positive changes, the literature supporting physiotherapy has improved, and because of these changes, physiotherapists are increasingly being referred to from the medical community. This element played a key role in the heterogeneity of the studies reviewed, making it difficult to compare a profession that is evolving to the medical model.

### Implications for Practice

To date, this is the first study that has reviewed the ability to screen and manage patients with musculoskeletal disorders through DAPT, showing the total autonomy and reliability of DAPT in the management of patients with musculoskeletal disorders. Recognizing DAPT as the appropriate case management process for MSDs could represent an opportunity for improving the quality of patients’ care [74]. This review adds to the body of evidence showing the superiority of DAPT compared to the traditional medical care model from an economic and patient satisfaction point of view. Regarding health outcomes, there is no difference between DAPT compared to the medical model. As previously demonstrated by other reviews, DAPT is safe, showing no adverse events on patients with musculoskeletal complaints or conditions.

## 6. Conclusions

In conclusion, DAPT appears to be equal to the physician-led model of care from the standpoint of health outcomes and safety, but it appears to be superior from the standpoint of economics and patient satisfaction. This is why DAPT needs to be implemented as it is a reliable, safe, effective, and economically favorable service which could play a key role in reducing healthcare costs.

## Figures and Tables

**Table 1:** Inclusion and exclusion criteria.

Inclusion Criteria:	Exclusion Criteria:
- Experimental primary study design that assesses the management accuracy, cost-effectiveness, safety, patient satisfaction, and health outcomes (assessed by clinical scales) of DAPT compared to traditional management (based on medical examination) within the public sector in patients with musculoskeletal disorders - Adult patients (>18 years) with MSDs - Studies written in English	- Studies that compare DAPT with another type of access to treatment (other than the medical model); or studies that compare another type of access to care, other than the DAPT, with the medical-centric model - Patients with disorders other than MSDs (i.e., neurological disorders) - Pediatric population - Non-experimental studies (protocols, narrative reviews, letters, clinical commentary) from the gray literature or secondary studies

**Table 2:** Search strategy for MEDLINE.

No	Searches	Results
1	(“Physical therapy” [All Fields] OR “physiotherapy” [All Fields])	144,912
2	(“Ambulatory Care” [All Fields] OR “Primary Health Care” [All Fields] OR “outpatient*” [All Fields] OR “primary care” [All Fields] OR “ambulator*” [All Fields] OR “delivery of health care” [All Fields])	713,311
3	(“Referral and Consultation” [All Fields] OR (“Referral and Consultation” [MeSH Terms] OR (“referral” [All Fields] AND “consultation” [All Fields]) OR “Referral and Consultation” [All Fields] OR “referral” [All Fields] OR “referrals” [All Fields] OR “referrer” [All Fields] OR “referrers” [All Fields]) OR “direct access” [All Fields] OR “dapt” [All Fields]) OR (“triage” [MeSH Terms] OR “triage” [All Fields] OR “triages” [All Fields] OR “triaged” [All Fields] OR “triaging” [All Fields]) OR “direct access physical therapy” [All Fields] OR “self-referral” [All Fields] OR “physical therapy direct access” [All Fields])	234,596
4	(“musculoskeletal disease*” [All Fields] OR “musculoskeletal disease” [All Fields] OR “musculoskeletal disorder*” [All Fields] OR “orthopedic disorder*” [All Fields])	25,095
5	(“cost-effectiveness” [All Fields] OR “effectiveness” [All Fields] OR “cost savings” [All Fields] OR (“economics” [MeSH Subheading] OR “economics” [All Fields] OR “cost” [All Fields] OR “costs and cost analysis” [MeSH Terms] OR (“costs” [All Fields] AND “cost” [All Fields] AND “analysis” [All Fields]) OR “costs and cost analysis” [All Fields]) OR “outcome*” [All Fields] OR (“prognosis” [MeSH Terms] OR “prognosis” [All Fields] OR “prognoses” [All Fields]))	4,901,682
6	1 AND 2 AND 3 AND 4 AND 5	70

**Table 3:** Population characteristics investigated in the current study.

Age (mean)	49 Years
Sex (%)	57% F–43% M
Type of MSDs (%)	Upper Limb 15% Spine 50% Lower Limb 25% Widespread/Mixed Pain 7% Rheumatological Disease 3%
Onset (%)	Acute (<6 weeks) 40% Sub-acute (6 weeks to 3 months) 25% Chronic (>3 months) 35%

**Table 4:** Type of specialization of the involved physiotherapists in DAPT.

Type of Specialization	Nr. of Studies
Post-graduate specialization/ doctorate or musculoskeletal certification	2 studies
1 day out for training; direct access to primary care and mentoring	1 study
From 6 to 28 years of experience	5 studies
At least 3 years of experience in primary care + at least 1 orthopedic manipulative physiotherapist specialization	4 studies
Extended or advanced scope practitioner	11 studies
Degree of physiotherapy	2 studies
Specialization not specified	3 studies

**Table 5:** DAPT management accuracy.

Parameter	Range	Mean
Patients independently screened by physiotherapist	69–97	80.6%
Surgical conversion rate	40–89.3%	67.4%

**Table 6:** Type of treatment prescribed.

	DAPT Mean (Min–Max)	Medical Model Mean (Min–Max)	Mean Difference
Imaging	21% (0–63%)	49% (27–86%)	28%
Medication	22.3% (8–50%)	63.5% (60–73.1%)	41.2%
Referral	9.3% (2.9–19.3%)	30% (14–40%)	20.7%

**Table 7:** Comparison between wage costs and treatment options and QALYs.

	DAPT Mean (Min–Max)	Medical Model Mean (Min–Max)	Mean Difference
Cost for episode of care	EUR 301.5 (255.55–628.24)	EUR 743.44 (498.38–988.51)	EUR 441.9

**Table 8:** Rate of presence to visit.

	DAPT Mean (Min–Max)	Medical Model Mean (Min–Max)	Mean Difference
Presence to visit	93.5% (90–97.1%)	87.5% (86–89%)	6%

**Table 9:** Time to triage/discharge the patient.

	DAPT Mean	Medical Model Mean	Mean Difference
Time to triage in minutes	108 min	148 min	40 min
Percentage of patients discharged within 4 h in primary care	93%	75%	18%
Number of sessions/days to discharge (Ankle MSDs)	5.6 sessions/ no difference	6.7 sessions/ no difference	1.1 sessions
Number of sessions/days to discharge (Knee MSDs)	6.3 sessions/ 49.7 days	9.1 sessions/ 60.2 days	2.8 sessions/ 10.5 days

**Table 10:** Percentage of sick leave prescriptions and number of sick leave days.

	DAPT Mean (Min–Max)	Medical Model Mean (Min–Max)	Mean Difference
Percentage of sick leave prescriptions	9% (3–15.1%)	12.16% (7.3–23.5%)	5%
Number of sick leave days prescribed	13.5 days (0–27 days)	50.5 days (26–75 days)	37 days

**Table 11:** DAPT safety.

Authors	Evaluation of DAPT Safety	Results
Peterson et al., 2021 [27]	Number of adverse events	none
Bishop et al., 2017 [32]	Number of adverse events	none
Salisbury et al., 2013 [34]	Number of adverse events	none
Bornhöft et al., 2019 [38]	Number of adverse events	none
Ojha et al., 2020 [46]	Number and type of adverse events	4 mild adverse events: 2/77 DAPT group 2/73 medical group Two patients in the medical group had an accidental fall at home, and unclear diagnosis of ankle pain at one-year and two patients in the DAPT group had side effects from an emergency room medication, and unclear diagnosis of low back pain.

**Table 12:** DAPT waiting times.

	DAPT Mean (Min–Max)	Physician-Led Care Model Mean (Min–Max)	Mean Difference
Waiting times in days	12.31 (3.55–26 days)	35.59 (28–57 days)	23.28 days
Primary care waiting time (min)	n.a.	n.a.	31 min

**Table 13:** Newcastle Ottawa Scale evaluation for case-control studies.

Reference	Selection of Case and Controls				Comparability of Cases and Controls	Exposure			Total
	**ITEM#1** **Is the case definition adequate**	**ITEM#2** **Representativeness of the cases**	**ITEM#3** **Selection of Controls**	**ITEM#4** **Definition of Controls** **Not Present at Start**	**ITEM#5** **Comparability of cases and controls on the basis of the** **design or analysis**	**ITEM#6** **Ascertainment of exposure**	**ITEM#7** **Same method of ascertainment for cases and controls**	**ITEM#8** **Non-Response Rate**	
Bird et al., 2016 [28]	✸		✸	✸	✸	✸	✸	✸	8/9
Bornhöft et al., 2015 [37]		✸		✸	✸		✸		5/9
Kooijman et al., 2013 [35]	✸	✸	✸	✸		✸	✸		6/9
Lankhorst et al., 2017 [44]	✸	✸	✸	✸		✸	✸	✸	7/9
Ludvigsson et al., 2012 [47]	✸	✸	✸	✸		✸	✸	✸	7/9
Mallet et al., 2014 [36]	✸	✸	✸	✸		✸	✸	✸	7/9
Oldmeadow et al., 2007 [18]	✸	✸	✸	✸	✸		✸	✸	7/9
Phillips et al., 2012 [43]	✸		✸	✸	✸				4/9
Szymanek et al., 2022 [48]	✸	✸	✸	✸	✸	✸	✸	✸	8/9

**Table 14:** Newcastle Ottawa Scale evaluation for cohort studies.

Reference	Selection of Cohorts				Comparability of Cohorts	Outcome			Total
	**ITEM#1** **Representativeness of the Exposed Cohort**	**ITEM#2** **Selection of the Non-Exposed**	**ITEM#3** **Ascertainment of Exposure**	**ITEM#4** **Demonstration that outcome of interest was not present at** **start of study**	**ITEM#5** **Comparability of cohorts on the basis of the design or analysis**	**ITEM#6** **Assessment of Outcome**	**ITEM#7** **Was follow up long enough for outcomes to occur**	**ITEM#8** **Adequacy of Follow Up of Cohorts **	
Caffrey et al., 2019 [26]	✸		✸	✸			✸	✸	5/9
Chang et al., 2018 [29]	✸		✸	✸				✸	4/9
Downie et al., 2019 [31]	✸		✸	✸			✸	✸	5/9
Kerridge-Weeks et al., 2016 [41]	✸		✸	✸		✸	✸	✸	6/9
Lyons et al., 2022 [19]	✸		✸	✸			✸	✸	4/9
O’Farrell et al., 2014 [40]	✸		✸	✸		✸	✸		5/9
Peterson et al., 2021 [27]	✸		✸	✸			✸	✸	5/9
Szymanek et al., 2022 [48]	✸		✸	✸			✸		4/9

## Data Availability

All data generated or analyzed during this study are included in this published study. Other information of this study are available from the corresponding author on reasonable request.
